# A Novel Lightweight Deep Learning Model for Boar Sperm Head Detection in Microscopic Images: YOLO11_SRP

**DOI:** 10.3390/ani16020258

**Published:** 2026-01-15

**Authors:** Mingchao Pan, Lin Gao, Zhendong Zhu, Yingqi Li, Mingkang Gao

**Affiliations:** 1 College of Automation and Electronic Engineering, Qingdao University of Science and Technology, Qingdao 266061, China; panmingchao2024@163.com (M.P.);; 2 College of Animal Science and Technology, Qingdao Agricultural University, Qingdao 266109, China; zzd2020@qau.edu.cn

**Keywords:** boar sperm, microscopic image, deep learning, YOLO, small-object detection

## Abstract

Accurate and quantitative detection of boar sperm heads is essential for breeding selection and reproductive management. Manual microscopic counting is time-consuming, labor-intensive, and prone to subjective bias, while existing computer-based algorithms often struggle to recognize sperm cells accurately when they overlap or move rapidly in high-magnification microscopic images. This study proposes a lightweight boar sperm detection model, YOLO11_SRP, designed to improve small-object recognition in complex microscopic scenarios. The model integrates a lightweight StarNet backbone, a rectangular self-calibration module for enhanced spatial feature modeling, and an additional low-level detection layer optimized for tiny targets. We evaluated the model on a boar sperm microscopic image dataset and compared it with the standard YOLO11s framework. The results show that YOLO11_SRP achieves an mAP@0.5 of 91.9%, representing a 13.9% improvement over YOLO11s, while simultaneously reducing parameters by 39% and computational cost by 14.1%. These findings demonstrate that YOLO11_SRP provides efficient and accurate sperm detection, supporting the development of efficient and reliable automated sperm analysis pipelines, in which sperm head detection serves as a fundamental preprocessing step.

## Simple Summary

Accurately counting boar sperm heads is important for selecting high-quality breeding animals and improving reproductive efficiency on farms. Traditionally, workers must observe sperm under a microscope and count them by hand, which requires considerable time, effort, and experience. However, existing algorithms often have difficulty recognizing sperm cells when they overlap or exhibit high motility in high-magnification images, leading to unreliable results. In this study, we developed a new lightweight computer-based method that can automatically identify boar sperm heads in microscope images more efficiently and more accurately. This method uses an improved image-recognition model that focuses better on tiny objects and learns patterns from thousands of sperm images. When we tested the system, it achieved higher detection accuracy than a commonly used model while also requiring less computing power. The proposed method is intended as a foundational step for automated sperm analysis, providing reliable sperm head detection that can support downstream analysis in practical breeding applications.

## 1. Introduction

Artificial insemination has become a cornerstone of modern swine production, enabling improved reproductive efficiency and supporting rapid genetic progress in boar breeding programs [1]. Accurate assessment of semen quality is essential for ensuring conception success and optimizing litter performance. Core indicators such as sperm motility, concentration, and morphology directly reflect boar reproductive capacity and influence insemination dosage, fertility outcomes, and herd renewal efficiency [2]. However, routine semen evaluation on commercial farms continues to rely heavily on manual microscopic inspection, a process that is labor-intensive, subjective, and highly dependent on operator expertise [3]. Although emerging multimodal optical platforms provide high-content information for individual sperm cells [4], their complexity and cost limit their adoption in large-scale swine production systems. Commercial Computer-Assisted Sperm Analysis (CASA) technologies offer semi-automated solutions [5,6,7], yet they remain constrained by hardware dependence, low compatibility with standard farm microscopes, and variable algorithmic performance across species and imaging conditions [8].

To address the limitations of manual assessment, earlier research explored classical image-processing techniques for sperm detection, tracking, and morphological quantification. These approaches—including connected-component labeling, Zernike moment feature extraction, and sperm-head localization—enabled initial progress in estimating motion characteristics or structural abnormalities [9,10,11,12]. However, their performance deteriorated under high-density, low-contrast, or overlapping microscopic conditions, as illustrated by failures in robust tracking or reliable sperm-head delineation when targets were extremely small and fast-moving [9,10]. More recent efforts coupled microfluidic imaging with deep learning-based detection to improve viability or motility analysis, but challenges such as chip reproducibility, imaging artifacts, and limited scalability persist [11].

With advancements in deep learning, particularly convolutional neural networks (CNNs), sperm detection research has shifted toward end-to-end object detection, segmentation, and classification frameworks. Early CNN-based methods demonstrated the feasibility of learning discriminative representations for spermatogenic cells and spermatozoa [12]. Subsequent studies applied YOLO, U-Net, or VGG-based models to human, bull, and boar sperm datasets, substantially enhancing detection accuracy and robustness. YOLOv5, for example, achieved a mean average precision (mAP) of 72.15% in sperm detection, although performance dropped significantly in dynamic or dense scenes and provided no direct information about sperm motility [13]. Other works focused on rare-sperm identification at low magnification [14] or segmentation of real sperm in video frames using modified U-Net architectures [15], yet these techniques struggled with inter-sperm boundary ambiguity and image noise. Deep learning models have also been deployed for detecting morphological abnormalities [16], recognizing healthy sperm heads [17], and enabling real-time detection in densely populated bull sperm videos [18]. Research on boar and bovine sperm morphology classification further confirmed the potential of CNN-based and multimodal systems to support automated fertility evaluation [19,20].

Beyond detection, deep learning has accelerated progress in quantitative-phase imaging, mobile-based analysis, and cell-part parsing via instance-aware networks, offering new avenues for label-free sperm morphology assessment [21,22,23]. Ensemble-based segmentation approaches have improved robustness in blurred or low-quality sperm images [24], while fully automated pipelines for sperm quality scoring demonstrated competitive performance relative to clinical standards [25]. Additionally, CNN-based motion prediction [26], machine learning-based motility estimation frameworks [27], and lightweight YOLO variants such as YOLOv5s-SA [28] expanded the scope of automated sperm analysis. More complex models, including YOLOv8E-TrackEVD for joint detection and tracking [29] and hybrid RCNN–tracking systems [30], provided enhanced spatiotemporal analysis at the cost of increased computational overhead.

Recent research trends have included GAN-based data enhancement for improved morphology classification [31], transformer-based spatiotemporal architectures for automated sperm tracking [32], category-aware morphological classification frameworks [33], and deep feature engineering using attention-augmented backbones [34]. Despite these developments, several unresolved challenges remain in boar sperm microscopic analysis: (1) boar sperm heads are extremely small, elongated, and densely distributed, complicating feature extraction for both one-stage detectors and segmentation networks; (2) dynamic microscopic environments—characterized by rapid motility, overlapping targets, and fluctuating illumination—introduce missed detections, false positives, and unstable predictions; and (3) many state-of-the-art models rely on heavy architectures that are difficult to deploy in resource-limited farm settings.

To address these limitations, this study proposes YOLO11_SRP, a lightweight deep learning framework specifically tailored for boar sperm head detection in microscopic images. Rather than introducing isolated architectural components, the proposed framework is designed from a task-driven perspective, in which the unique visual characteristics of sperm microscopy—such as dense object distribution, weak boundaries, directional structures, and extremely small target sizes—are explicitly considered in the network design.

Specifically, YOLO11_SRP integrates three complementary adaptations: a lightweight StarNet backbone to improve feature extraction efficiency under dense and low-contrast conditions, a Rectangular Self-Calibration Module (RCM) to enhance directional sensitivity and foreground–background discrimination in complex microscopic fields, and a P2 small-object detection layer to preserve high-resolution shallow features critical for accurate perception of minute sperm heads. The novelty of the proposed method lies not in the individual components themselves, but in their coordinated adaptation and integration for sperm microscopy, forming an architecture that better aligns with the domain-specific challenges of microscopic semen analysis.

Experimental results demonstrate that YOLO11_SRP improves mAP@0.5 by 13.9% over YOLO11s while reducing parameters by 39% and computational cost by 14.1%, achieving a favorable balance between accuracy, robustness, and lightweight deployment. These findings highlight the potential of the proposed model to support scalable and high-efficiency computer-assisted semen analysis in modern swine production.

## 2. Materials and Methods

### 2.1. Overall Architecture

**Figure 1:**
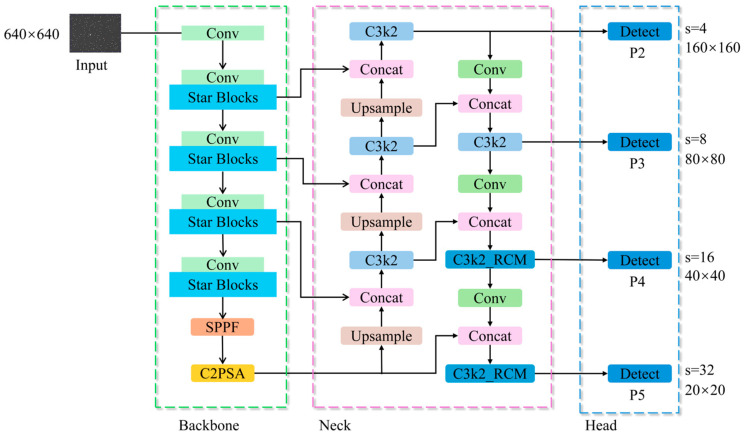
Overall architecture of the YOLO11_SRP model.

The proposed YOLO11_SRP model is developed based on the YOLO11s framework and is specifically optimized for the challenges inherent in boar sperm detection, including extremely small object size, high target density, and complex microscopic backgrounds. As illustrated in Figure 1, the overall architecture consists of three major components: the Input, Backbone, and Detection Head.

In the input stage, microscopic images are normalized to ensure numerical stability and a consistent intensity distribution.

In the feature extraction stage, the lightweight StarNet architecture is adopted to replace the conventional CSPDarknet backbone. This design choice is motivated not only by computational efficiency, but also by the requirement for stable feature representation under dense target distributions, which are characteristic of sperm microscopy but less prominent in natural-image detection tasks.

In the feature fusion and detection stage, a P2 small-object detection layer is introduced to enhance shallow feature representations and alleviate the loss of fine-grained spatial details caused by repeated downsampling. Meanwhile, the Rectangular Self-Calibration Module (RCM) is embedded into higher-level feature fusion layers to explicitly model directional and contextual dependencies arising from sperm alignment, local motion patterns, and dense spatial arrangements in microscopic images. The coordinated integration of the P2 detection layer and RCM reflects a task-driven architectural design aimed at jointly addressing scale sensitivity and contextual ambiguity in sperm head detection, rather than a simple accumulation of independent network components.

Finally, detection is performed at four feature scales—P2, P3, P4, and P5—enabling accurate identification of sperm heads across different resolutions while maintaining computational efficiency.

### 2.2. Replacement of the Backbone with StarNet

The backbone of YOLO11s relies on conventional convolutions and C3k2 modules, which extract features through stacked convolutional layers. While effective, standard convolutions incur high computational and parameter costs. To address this limitation, StarNet is introduced as a lightweight backbone replacement in YOLO11_SRP [35].

**Figure 2:**
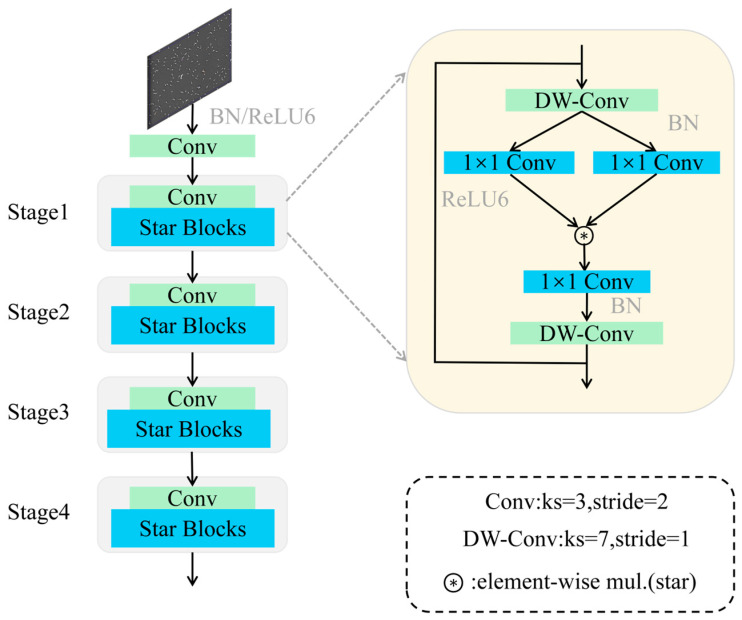
Network architecture of StarNet.

StarNet is an efficient neural architecture based on the star operation, which maps low-dimensional features into high-dimensional nonlinear feature spaces through element-wise multiplication. This design significantly enhances representational capacity while maintaining computational efficiency. The structure of StarNet is shown in Figure 2.

The model begins with an initial convolutional layer for primary feature extraction, followed by batch normalization (BN) and a ReLU6 activation function to accelerate convergence and strengthen nonlinear expression. Deeper and more complex features are progressively extracted through four stages, each comprising one convolutional layer and multiple Star Blocks, which consist of two depthwise separable convolutions and three channel-wise transformation layers implemented via point-wise (1 × 1) convolutions.

The key feature of StarNet is the fusion of two linear transformations through element-wise multiplication, generating an enriched high-dimensional feature space. This operation resembles a polynomial kernel function, enabling high-dimensional mapping without increasing network width or channel count. Consequently, StarNet enhances feature extraction while significantly reducing computational overhead, achieving a lightweight yet expressive backbone for YOLO-based detection frameworks.

By leveraging the star operation, StarNet implicitly captures high-dimensional nonlinear features while performing computation in a low-dimensional space. This approach provides expressive feature modeling without additional computational cost, similar to classical kernel methods. The combination of compact structure, low latency, and strong representational power makes StarNet an ideal backbone for lightweight YOLO models, facilitating high-performance detection in resource-constrained environments.

### 2.3. Structure and Principle of the RCM

**Figure 3:**
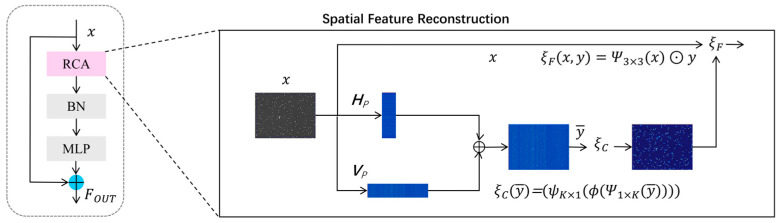
Network architecture of the RCM.

To address the challenges in boar sperm detection, such as unclear foreground–background separation, dense target distribution, ambiguous boundaries, insufficient localization accuracy, and multi-scale object variation, this study incorporates the RCM into the YOLO11_SRP framework [36]. As illustrated in Figure 3, RCM consists of four main components: Rectangular Self-Calibration Attention (RCA), a shape self-calibration function, Batch Normalization (BN), and a Multi-Layer Perceptron (MLP). Here, the MLP is implemented using point-wise (1 × 1) convolutional layers applied directly to convolutional feature maps, without flattening or true fully connected operations.

The Rectangular Self-Calibration Module (RCM) first generates multi-scale feature maps through a sequence of convolutional and down-sampling operations, providing more discriminative representations for the subsequent sperm detection task. Within the RCM, the incorporated Receptive Context Attention (RCA) models the dependencies among different spatial regions of the feature maps and dynamically adjusts feature responses to highlight key contextual information relevant to foreground sperm targets.

During the pyramidal context extraction stage, multiple RCM units progressively process the fused features to integrate multi-scale spatial cues, suppress background interference, and enhance structurally salient patterns. Leveraging large-kernel convolutions for adaptive adjustment of attention shapes, the module better conforms to the actual morphology and motion characteristics of sperm heads, thereby improving localization accuracy for foreground targets. Meanwhile, depthwise convolutions further refine local details, enabling the model to exhibit higher stability and robustness when detecting low-contrast, small-sized sperm objects and objects with blurred boundaries.

**Figure 4:**
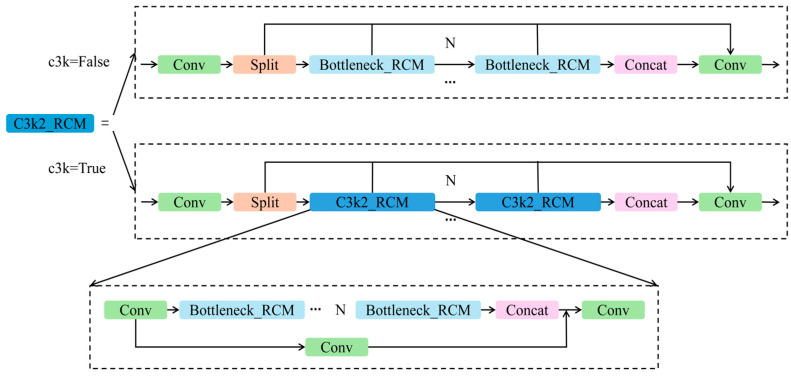
Architecture of the C3k2_RCM module.

In YOLO11s, the C3k2 module plays a critical role in adjusting receptive field sizes and capturing multi-scale features. The proposed C3k2_RCM hybrid module are deployed in the neck at P4–P5 levels, illustrated in Figure 4, integrates RCM into the original C3k2 structure. This design enables RCM to contribute rich multi-scale contextual modeling, thereby improving feature fusion across different resolutions and substantially enhancing YOLO11s’ detection performance under complex microscopic environments.

### 2.4. Design of the P2 Small-Object Detection Layer

**Figure 5:**
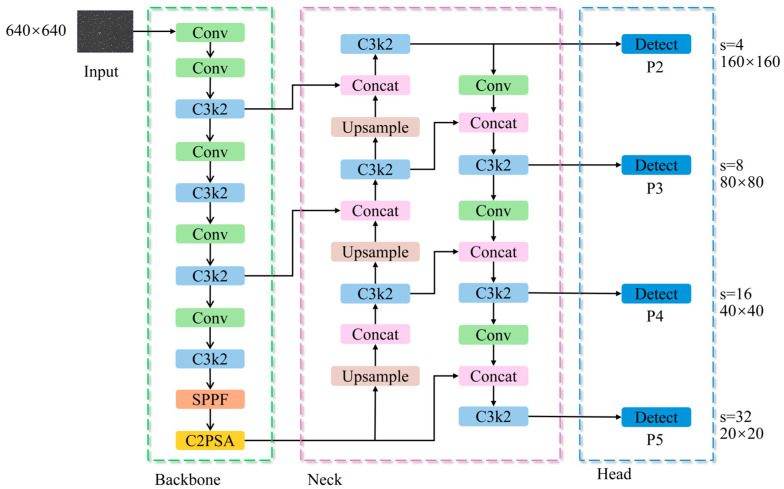
Structure of YOLO11s after adding P2.

In the original architecture of YOLO11s, the feature output layers consist of three detection scales, P3, P4, and P5, corresponding to feature maps downsampled by factors of 8, 16, and 32, respectively. While this configuration performs well for medium- and large-sized objects, it is suboptimal for sperm cells, which are extremely small, elongated, and densely distributed. The high downsampling ratios lead to loss of fine-grained boundary information, thereby degrading detection accuracy. To address this limitation, an additional small-object detection layer, P2, is incorporated into the detection head of YOLO11s to extract higher-resolution features with a 4× downsampling rate, as illustrated in Figure 5. The P2 layer is directly connected to the shallow feature maps generated in the early stages of the backbone, where feature integration is performed through the C3k2 module, followed by concatenation with the upsampled high-level semantic features during the feature fusion process. Compared with deeper layers, the P2 feature map retains richer texture and boundary cues due to its higher spatial resolution, thereby substantially enhancing the model’s capability for accurate localization and refined delineation of small boar sperm targets.

## 3. Results

### 3.1. Dataset Construction

Since no publicly available boar sperm image dataset currently exists, this study constructed a dedicated boar sperm detection dataset using professional biological microscopic imaging equipment. All raw images were acquired from fresh boar semen samples and captured using negative phase contrast microscopy at a magnification of 200×. The dataset includes images at two different resolutions: images from dense scenes have a resolution of 776 × 584 pixels, whereas images from sparse scenes have a resolution of 1920 × 1080 pixels. The typical pixel size of boar sperm heads in the images is approximately 9 × 11 pixels in dense scenes and 37 × 31 pixels in sparse scenes.

The semen samples were obtained by collecting a homogenized mixture of ejaculates from nine Duroc boars. The dataset contains a total of 843 fully annotated images, with dense and sparse scene images accounting for approximately 43% and 57% of the dataset, respectively. On average, dense-scene images contain 155 boar sperm heads, while sparse-scene images contain 46 boar sperm heads. The dataset encompasses a wide range of sperm densities, motility states, and imaging conditions, thereby providing sufficient diversity to support model training and performance evaluation.

The dataset was randomly divided into training, validation, and test sets at a ratio of 8:1:1, corresponding to 675, 84, and 84 images, respectively. As all images were derived from the same pooled semen sample, no explicit constraints were imposed to prevent images from the same source appearing across different subsets, and the data split was performed in a fully random manner.

### 3.2. Experimental Environment and Evaluation Metrics

All experiments were conducted on an NVIDIA GeForce RTX 3090 GPU using the PyTorch deep learning framework. The environment configuration consisted of Python 3.8, PyTorch 2.0.0, and CUDA 11.8. To enhance detection performance and prevent convergence to local minima, the Stochastic Gradient Descent (SGD) optimizer is adopted, with an initial learning rate of 0.01, momentum of 0.937, batch size of 16, and 125 training epochs.

Evaluation metrics included Precision (P), Recall (R), mean Average Precision (mAP), model size (MB), number of parameters (Params), and floating-point operations per second (FLOPs). The precision (P), recall (R), and mean average precision (mAP) are calculated according to Equations (1)–(4):

(1) $$P=\frac{TP}{TP+FP}$$

(2) $$R=\frac{TP}{TP+FN}$$

(3) $$mAP=\frac{\sum_{i=1}^{n}AP_{i}}{n}$$

(4) $$AP=\int_{0}^{1}P\left(R\right)dR$$

where TP denotes true positives, FP false positives, FN false negatives, and AP_i_ the average precision of class i. The metric mAP@0.5 refers to the mAP computed at an IoU threshold of 0.5. FLOPs reflect the computational complexity of the model, affecting training time, inference speed, and deployment efficiency.

### 3.3. Ablation Study

In this study, YOLO11s is used as the baseline model, and a series of ablation experiments are conducted under identical training settings to evaluate the contribution of each proposed modification. The results are summarized in Table 1.

- In Group 1, the results represent the baseline performance of YOLO11s.
- In Group 2, after replacing the original backbone with the lightweight StarNet backbone, precision and recall increased by 1.2% and 11.3%, respectively, while mAP@0.5 remained nearly unchanged. Meanwhile, the parameter count and FLOPs were reduced by 42.17% and 47.42%, respectively. These results indicate that StarNet can substantially reduce model complexity while preserving sufficiently discriminative feature representations for sperm head detection.
- In Group 3, substituting the original C3k2 module with the improved C3k2_RCM module led to increases of 2.2% in precision and 1.7% in recall, along with a moderate improvement in both mAP@0.5 and mAP@0.5:0.95. Although this modification slightly increased parameters and computational cost, it effectively enhanced contextual feature modeling and improved localization quality under stricter IoU criteria.
- In Group 4, introducing the P2 detection head resulted in substantial improvements across all evaluation metrics, with precision, recall, mAP@0.5, and mAP@0.5:0.95 increasing by 9.9%, 16.1%, 11.5%, and 12.4%, respectively. This demonstrates that incorporating a high-resolution detection layer significantly benefits both small-object sensitivity and bounding box regression accuracy in dense microscopic scenes.
- In Group 5, when the StarNet backbone and the C3k2_RCM module were introduced simultaneously, the number of parameters and FLOPs were reduced by 36.2% and 36.5%, respectively, accompanied by a slight improvement in precision. However, both recall and mAP values were lower than those achieved when each component was applied independently. From a feature-level perspective, StarNet suppresses redundant shallow activations during structural compression, which weakens fine-grained spatial representations. As the effectiveness of the C3k2_RCM module relies on informative low-level features, this degradation limits its ability to recover small or partially occluded sperm heads, leading to reduced recall and localization accuracy, while the improved consistency of high-level semantics helps maintain precision.
- In Group 6, where the C3k2_RCM module and the P2 detection head were combined without backbone compression, detection performance remained comparable to that of Group 4, with consistently high mAP@0.5 and mAP@0.5:0.95 values. This indicates that contextual enhancement and high-resolution detection can be mutually beneficial when sufficient shallow feature capacity is preserved.
- In Group 7, after introducing the StarNet backbone together with the P2 detection head, all performance metrics were slightly lower than those of Group 4 and Group 6o, although still markedly higher than the baseline. This suggests that backbone compression alters the statistical distribution of shallow feature maps, partially weakening the ability of the P2 detection head to exploit fine-grained spatial cues, which in turn affects recall and bounding box regression quality.
- In Group 8, when the StarNet backbone, C3k2_RCM module, and P2 detection head were integrated simultaneously, precision, recall, mAP@0.5, and mAP@0.5:0.95 all reached their optimal values among all experimental groups. This demonstrates that a complementary feature hierarchy is formed through the coordinated design of the three components. Specifically, StarNet provides a lightweight and noise-suppressed feature foundation, the C3k2_RCM module enhances semantic consistency and contextual discrimination at intermediate and high levels, and the P2 detection head compensates for the loss of fine spatial details by reinforcing shallow-scale representations. The synergistic optimization across feature compression, contextual calibration, and small-object sensitivity enables balanced multi-scale perception and yields the best overall detection performance.

### 3.4. Comparative Experiments

To thoroughly evaluate the effectiveness and superiority of the proposed YOLO11_SRP model, eight representative YOLO-based detectors were selected as comparison baselines: YOLO11s, YOLOv6s, YOLOv8s, YOLOv9s, YOLOv10s, YOLOv12s, YOLOv13s, and Hyper-YOLO. All compared models were retrained from scratch on the same boar sperm dataset using identical training hyperparameters (SGD optimizer, initial lr = 0.01, batch size = 16, 125 epochs) to ensure fair comparison. Results are shown in Table 2.

In addition to YOLO11-based variants, several recently proposed YOLO versions, including YOLOv12s [37] and YOLOv13s [38], were also included for comparative evaluation. YOLOv12 is an attention-centric real-time object detector proposed by Tian et al., which emphasizes global feature interaction through attention-driven architectural design. YOLOv13 further introduces a hypergraph-enhanced adaptive visual perception mechanism to improve relational modeling among visual elements. As these models have been proposed only recently and are not yet widely standardized in microscopic object detection benchmarks, they are evaluated in this study as comparative reference methods. To ensure a fair comparison, all models are trained from scratch on the same dataset using identical training protocols, hyperparameter settings, and evaluation metrics.

The comparative results in Table 2 demonstrate that YOLO11_SRP achieves the best overall detection performance among all evaluated models. Relative to the baseline YOLO11s, the proposed model exhibits substantial improvements, with precision increased by 10.7%, recall by 19.0%, and mAP@0.5 by 13.9%. Notably, YOLO11_SRP also improves mAP@0.5:0.95 from 54.4% to 67.9%, indicating a substantial enhancement in localization accuracy under stricter IoU criteria. This reflects a markedly enhanced capability to detect small and densely distributed sperm targets. Even when compared with high-performing detectors such as YOLOv9s and YOLOv12s, YOLO11_SRP maintains a clear performance advantage in both mAP@0.5 and mAP@0.5:0.95, confirming that the proposed multi-module enhancement strategy effectively strengthens feature representation and bounding box regression accuracy in complex microscopic environments.

In terms of model complexity, YOLO11_SRP contains only 5.74 million parameters, 18.3 GFLOPs, and a 12.0 MB weight file, all notably lower than YOLO11s and most contemporary YOLO variants, except for Hyper-YOLO [39]. However, despite Hyper-YOLO’s lightweight design, its accuracy remains significantly lower, indicating that YOLO11_SRP achieves a more favorable balance between detection performance and computational efficiency. These results collectively demonstrate that YOLO11_SRP not only delivers state-of-the-art accuracy for sperm head detection but also retains the lightweight characteristics necessary for practical deployment in real-world sperm analysis systems.

### 3.5. Qualitative Visualization Analysis

**Figure 6:**
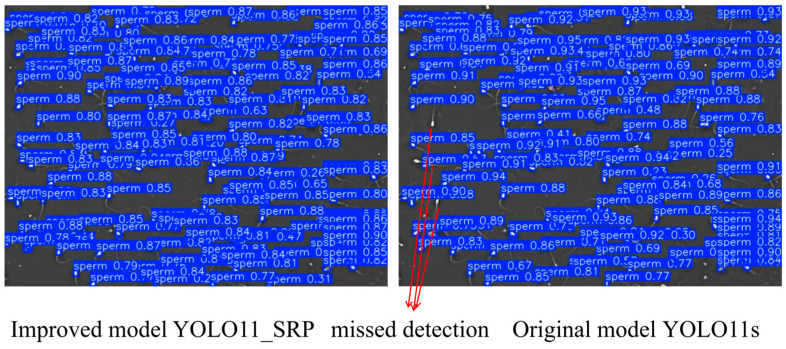
Dense Scene Detection Performance Comparison.

**Figure 7:**
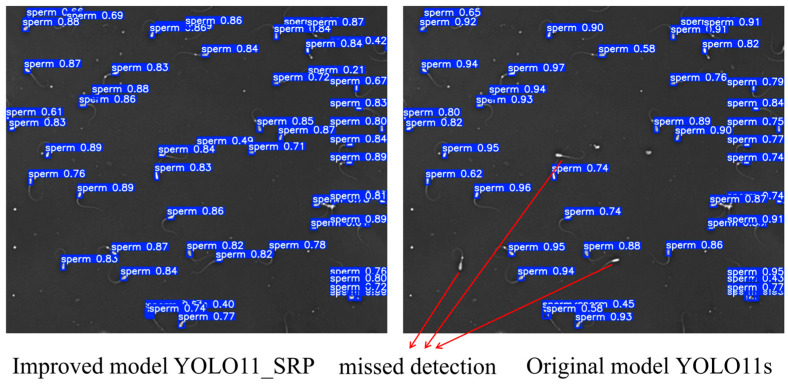
Sparse Scene Detection Performance Comparison.

To further evaluate the practical detection performance of YOLO11_SRP, representative samples from dense and sparse sperm scenes were selected for qualitative comparison with the baseline YOLO11s. The visual results are shown in Figure 6 and Figure 7.

From the results, it can be observed that YOLO11s exhibits obvious target missed detection in both scenarios, while YOLO11_SRP demonstrates superior detection performance in both cases: in complex regions with highly dense sperm, the improved model can more accurately distinguish closely adjacent targets and significantly reduce missed detections; in scenarios with relatively sparse sperm distribution, the model also outputs higher confidence and achieves clearer and more complete bounding box localization. By contrast, YOLO11s has obvious target omissions in dense regions and also suffers from issues such as unstable detection in sparse scenarios. The aforementioned visualization results further indicate that YOLO11_SRP possesses stronger robustness and detection reliability in practical applications, which verifies the effectiveness of the structural improvements proposed in this study.

## 4. Discussion

In this study, an improved YOLO-based framework was developed for automated detection of boar sperm heads in microscopic images, with the aim of addressing the limitations of manual evaluation and conventional computer-assisted sperm analysis (CASA) systems. The experimental results demonstrate that the proposed method achieves robust and accurate performance under challenging conditions, including high sperm density, small target size, low contrast, and frequent object overlap. The following discussion interprets these findings in relation to previous studies, highlights the contribution of the proposed model design, and outlines current limitations and future research directions.

### 4.1. Performance Interpretation and Comparison with Previous Studies

Compared with traditional image processing–based approaches, such as connected component analysis or handcrafted feature extraction methods (e.g., Zernike moments), the proposed deep learning–based detector shows a clear advantage in detection robustness and generalization ability. Earlier studies relying on classical techniques have reported acceptable performance only under controlled imaging conditions, while experiencing significant degradation in dense or noisy scenes [9,10,11,12]. In contrast, the proposed method exhibits consistently balanced precision and recall across different microscopic samples in the test set, suggesting improved robustness under the experimental conditions considered in this study.

When compared with existing deep learning–based sperm detection methods, including YOLOv5- and YOLOv8-based models [28,29], the proposed framework exhibits improved detection accuracy, particularly for small and densely clustered sperm heads. This improvement suggests that generic object detection architectures, although effective for natural images, require task-specific adaptations to cope with the unique visual characteristics of sperm microscopy, such as weak boundaries, rapid motion, and frequent occlusion. The experimental results support the hypothesis that targeted architectural modifications are essential for reliable sperm detection in high-density microscopic environments.

### 4.2. Contribution of Model Design to Detection Performance

Unlike generic object detection tasks, sperm microscopy is characterized by dense target distributions, extremely small object sizes, and pronounced directional structures. The proposed model demonstrates that explicitly aligning network architecture with these domain-specific properties is critical for achieving reliable detection performance.

In particular, the inclusion of a dedicated small-object detection layer enhances the model’s sensitivity to sperm heads, whose projected area occupies only a small fraction of the image. This design choice effectively mitigates the scale mismatch problem commonly encountered in standard YOLO architectures.

Second, the rectangular self-calibration module plays a critical role in improving feature representation. By integrating receptive context attention, the RCM explicitly models dependencies between spatial regions in the feature map, allowing the network to emphasize contextually relevant foreground information while suppressing background noise. The adaptive attention mechanism, combined with large-kernel convolutions, enables flexible adjustment of the attention shape, which better aligns with the elongated and irregular appearance of sperm heads under different imaging conditions. As a result, the model demonstrates enhanced localization accuracy and reduced false detections in cluttered scenes.

Furthermore, the use of depthwise and lightweight convolutional operations ensures that these performance improvements do not come at the cost of excessive computational overhead. This balance between accuracy and efficiency is particularly important for practical deployment in laboratory or on-farm settings, where stable inference under limited computational resources is often required.

### 4.3. Comparison with CASA and Microfluidic-Based Systems

From an application perspective, the proposed method offers several advantages over conventional CASA systems. Traditional CASA relies heavily on predefined thresholds and parameter tuning, which can introduce subjectivity and limit robustness across different laboratories or imaging setups [5,7]. By contrast, the proposed deep learning–based approach learns discriminative features directly from data, thereby reducing dependence on manual parameter adjustment and operator experience.

Recent studies have explored the integration of microfluidic chips with deep learning techniques to assess sperm quality or viability [11]. While such systems can improve measurement consistency, they often require specialized hardware and suffer from issues related to chip reproducibility, imaging artifacts, and limited scalability. The proposed framework operates directly on standard microscopic videos without additional hardware modifications, making it more accessible and easier to integrate into existing semen evaluation workflows. It should be emphasized that the proposed framework is not intended to serve as a complete replacement for conventional CASA systems. Instead, it focuses on accurate sperm head detection as a foundational step in automated semen analysis. The detection results can be further integrated with downstream modules, such as sperm tracking, motility assessment, or morphological analysis, to form a more comprehensive evaluation pipeline.

### 4.4. Limitations of the Present Study

Despite the promising results, several limitations should be acknowledged. First, the dataset used in this study was collected under relatively controlled laboratory conditions. Variations in microscope type, illumination settings, or sample preparation protocols may affect the generalization performance of the model. Additional multi-center datasets will be required to further validate the robustness of the proposed method.

Second, the current work focuses primarily on sperm head detection, without explicitly modeling tail morphology or detailed motility trajectories. Although accurate head detection is a prerequisite for reliable counting and tracking, comprehensive sperm quality assessment also requires quantitative analysis of motion patterns and morphological abnormalities. Integrating temporal information and multi-object tracking strategies may further enhance the analytical capabilities of the system.

### 4.5. Implications and Future Research Directions

Overall, the proposed framework provides an effective and practical solution for automated boar sperm head detection, serving as a core component rather than a complete replacement of existing CASA systems. By combining task-specific architectural design with lightweight computation, the method has the potential to serve as a core module in next-generation intelligent CASA systems.

Future research will focus on extending the current framework by incorporating multi-object tracking algorithms for integrated sperm trajectory analysis and motility assessment, as well as adding instance segmentation or classification heads for detailed morphology evaluation. These extensions will enable the quantification of key motility parameters beyond simple counting, such as curvilinear velocity (VCL), straight-line velocity (VSL), average path velocity (VAP), linearity (LIN), straightness (STR), wobble (WOB), amplitude of lateral head displacement (ALH), and beat-cross frequency (BCF), which are critical indicators of progressive and hyperactivated motility essential for fertilization success.

Similarly, morphology analysis will target the classification and proportion of defects in the head (e.g., abnormal shape or size), midpiece (e.g., distal midpiece reflex, cytoplasmic droplets), and tail (e.g., bent, coiled, or broken tails), along with overall normal morphology percentage. Such advancements, combined with validation in large-scale on-farm and clinical applications, may contribute to more objective, efficient, and standardized semen quality evaluation in modern animal breeding and reproductive management.

## 5. Conclusions

This study proposes a lightweight boar sperm detection model, YOLO11_SRP, for microscopic scenarios. By integrating three key improvements, the StarNet backbone network, the Rectangular Self-Calibration Module, and a newly added detection layer optimized for small targets, this model effectively addresses the detection challenges such as the tiny size of sperm head targets, dense overlap, and complex microscopic backgrounds. Experimental results show that compared with the baseline model YOLO11s, both detection accuracy and inference efficiency are improved. Compared with other similar methods, YOLO11_SRP still demonstrates stable and reliable detection performance in microscopic images with high-density sperm distribution and multi-interference backgrounds, thus possessing good engineering application value and promotion potential.

## Figures and Tables

**Table 1:** Ablation experiment results.

Model	YOLO11s	StarNet	C3k2_RCM	P2	P/%	R/%	mAP@0.5/%	mAP@0.5:0.95/%	Params/M	FLOPs/G	Weights/MB
1	√	×	×	×	82.4	66.8	78	54.4	9.41	21.3	19.2
2	√	√	×	×	83.6	78.1	78.1	52.3	5.44	11.2	11.2
3	√	×	√	×	84.6	68.5	80.6	56.4	9.57	21.5	19.5
4	√	×	×	√	92.3	82.9	89.5	66.8	9.56	28.6	19.6
5	√	√	√	×	83.9	67	78.9	53.6	5.60	11.4	11.6
6	√	×	√	√	92.2	82.8	89.0	66.9	9.72	28.8	20
7	√	√	×	√	92.2	81.3	88.5	64.3	5.59	18.1	11.7
8	√	√	√	√	93.1	85.8	91.9	67.9	5.74	18.3	12.0

Note: “√” denotes that the module is enabled, and “×” denotes that the module is not enabled.

**Table 2:** Comparison experiment results.

Model	P/%	R/%	mAP@0.5/%	mAP@0.5:0.95/%	Params/M	GFLOPs/G	Weights/MB
YOLO11s	82.4	66.8	78	54.4	9.41	21.3	19.2
YOLOv6s	85.8	70.2	81.4	53.1	16.30	44.0	32.8
YOLOv8s	84.1	65.3	77	54.4	11.12	28.4	22.5
YOLOv9s	86.2	72.8	84.1	58.1	7.17	26.7	15.2
YOLOv10s	83.8	69	81.6	55.4	7.21	21.4	16.5
YOLOv12s	86.9	69.7	82.4	57.6	9.23	21.2	18.9
YOLOv13s	87	68.8	82.1	56.7	9.00	20.7	18.6
Hyper-YOLO	85.8	68.5	81.3	54.0	3.94	10.8	8.2
YOLO11_SRP	93.1	85.8	91.9	67.9	5.74	18.3	12.0

## Data Availability

The datasets presented in this article are not readily available because the datasets are part of an ongoing study. Requests to access the datasets should be directed to Lin Gao (Qingdao University of Science and Technology), E-mail: gaolin0619@126.com.
